# Clinical Comparison of *Fusarium* Keratitis according to the Initial Potassium Hydroxide (KOH) Smear: A Retrospective Study in South Korea

**DOI:** 10.1155/2022/9106429

**Published:** 2022-11-18

**Authors:** Jong Ha Kim, Hyeon Jeong Yoon, Kyung Chul Yoon, In Cheon You

**Affiliations:** ^1^Department of Ophthalmology, Jeonbuk National University Medical School, Research Institute of Clinical Medicine of Jeonbuk National University-Biomedical Research Institute of Jeonbuk National University Hospital, Jeonju, Republic of Korea; ^2^Department of Ophthalmology, Chonnam National University Medical School and Hospital, Gwangju, Republic of Korea

## Abstract

**Purpose:**

This study aimed to compare predisposing factors, clinical characteristics, treatment, and prognosis of *Fusarium* keratitis according to the result of the initial potassium hydroxide (KOH) smear.

**Methods:**

This is a retrospective study of cases with *Fusarium* keratitis between January 2000 and December 2019 at two tertiary hospitals in South Korea. Patients were divided into two groups depending on the KOH smear result (KOH-positive and KOH-negative group), and its clinical factors were analyzed.

**Results:**

Among 319 fungal keratitis, seventy-nine cases were identified with *Fusarium* keratitis. Forty-seven cases (59.5%) were negative in the initial KOH smear prior to their diagnosis. The most common predisposing factor for *Fusarium* keratitis was ocular trauma (55.7%). There were no significant differences in sex, occupation, ulcer size or shape, hypopyon, and initial visual acuity between the two groups. Differences were observed between the KOH-positive group and the KOH-negative group in terms of deep corneal infiltration (50.0% vs. 78.7%, *p*=0.008) and evisceration treatment (3.1% vs. 25.5%, *p*=0.008). The delayed time to initiate antifungal eye drops was longer in the KOH-negative group (1.13 ± 0.49 vs. 3.93 ± 4.89, *p*=0.002). Only the KOH-negative group combined bacterial infection. The significant risk factors for poor clinical outcomes were the central corneal lesion (odds ratio (OR) 3.50, *p*=0.047), a large ulcer size (size ≥ 7.5 mm^2^) (OR 4.98, *p*=0.009), and endothelial plaque (OR 7.00, *p*=0.031).

**Conclusion:**

Initial KOH-negative patients often needed evisceration and had worse final visual outcomes. The delay of prompt initiation of antifungal treatment and combined bacterial infection result in a poor prognosis. This study highlights the initial KOH effect on early diagnosis and early treatment of *Fusarium* keratitis.

## 1. Introduction

*Fusarium* species (spp.) are ubiquitous filamentous fungi that are present in water, soil, and plant roots and are one of the most commonly reported organisms causing keratomycosis [1–3]. The clinical diagnosis of *Fusarium* keratitis is challenging, and management is often difficult due to its insidious onset, diversity of clinical presentation, deep corneal stromal penetrating capabilities resulting in protracted response to medical therapy, prolonged microbiological identification time, and the emergence of broad resistance to most antifungals currently available [3–5]. Additionally, antifungal treatment may be delayed due to primary therapy with an antibacterial agent or antiviral agent [3]. *Fusarium* keratitis might progress to a deep extensive infection with perforation and endophthalmitis, which ultimately may lead to monocular blindness [1, 6, 7]. Several predisposing factors for the development of *Fusarium* keratitis include ocular trauma, particularly from vegetative or soil-contaminated objects, prior corneal surgery, previous chronic ocular surface disease, contact lens wear, and immunosuppressive disease or medication [1, 4, 5, 8].

The epidemiological pattern of fungal keratitis varies across geographic regions and climatic conditions [9]. *Fusarium* and *Aspergillus* species are more common in tropical and subtropical regions such as India and Mexico [9, 10]. In these countries, corneal epithelial defects caused by ocular trauma are the main predisposing factor for *Fusarium* keratitis [5, 9, 10]. However, in patients using contact lenses, *Fusarium* keratitis has also become a problem in urban areas with temperate climates. From 2005 to 2006, contact lens-associated*Fusarium* keratitis outbreak was reported in Hong Kong, Singapore, France, and the United States [11–16].

The traditional diagnostic method of fungal keratitis is mostly to stain and culture corneal specimens after scraping the ulcerative tissue. The more accurate diagnosis of keratitis mainly relies on a fungal culture. However, the fungal culture requires longer time to identify pathogens, and generally, the cultivation time is 1–2 weeks [17]. The potassium hydroxide (KOH) smear is one of the most commonly used methods for early direct microscopic detection of fungal filaments. One study reported that 10% KOH preparation was of marked importance, with a sensitivity and specificity value of 99.3% and 99.1%, respectively, for detection of fungal filaments [18]. The KOH smear is recommended in every clinic for initial rapid diagnosis of infective keratitis including fungal species. Infective keratitis can be reduced by early identification of microbial pathogens, which provides a prerequisite for early treatment of keratitis [17].

Therefore, we conducted a comparative study of cases with microbiologically proven *Fusarium* keratitis according to the result of the initial KOH smear at two tertiary hospitals in Jeolla Province, South Korea. Demography, disease history, clinical manifestations, treatment outcomes, and prognosis of patients have been analyzed retrospectively for the past 20 years (2000–2019).

## 2. Materials and Methods

We used a retrospective, linear, and comparative study design. We retrospectively reviewed medical records of all culture-proven cases of *Fusarium* keratitis that were treated at the Department of Ophthalmology of Jeonbuk National University Hospital and Chonnam National University Hospital in South Korea between January 2000 and December 2019. All patients underwent slit-lamp examinations and detailed clinical evaluations, including the smear and culture. The inclusion criteria were clinical evidence of fungal keratitis, such as a positive fungal culture from a corneal specimen, positive identification of fungal elements on the KOH smear, and histopathology showing the presence of fungal elements. The exclusion criteria were the cases that did not receive antifungal therapy, such as herpetic viral keratitis, Acanthamoeba infections, and noninfectious corneal diseases. This study was approved by the Jeonbuk National University Medical School Institutional Review Board and Ethics Committee. When studying data, patients were divided into two groups depending on the initial KOH smear: those with identification of fungal elements on the 10% KOH smear before the diagnosis of fungal keratitis on the culture as a KOH-positive group and those with no identification of fungal elements on the KOH smear before diagnosis as a KOH-negative group.

Baseline epidemiology, predisposing factors, clinical manifestations, and treatment were evaluated and compared between the KOH-positive and KOH-negative groups. The epidemiologic characteristics included sex, laterality, occupation, residence area, and systemic diseases. Information on the past history and predisposing factors was focused on the presence of ocular trauma, use of contact lenses, pre-existing chronic ocular surface disease, and prior corneal surgery. The initial clinical manifestations included the location, shape of ulcer, depth of infiltration, presence of feathery margin, satellite lesion, endothelial plaque, and hypopyon, size of corneal lesion, and presenting best-corrected visual acuity as determined using Snellen test. The size of the infiltrate was measured by multiplying two linear dimensions and the longest linear diameter and its largest possible perpendicular within the confines of the epithelial defect using a slit lamp [19]. The location of ulceration was categorized into three zones, central, paracentral, and peripheral. The depth of infiltration was classified into superficial (anterior < 2/3 of the total corneal thickness) and deep (≥2/3 of the total corneal thickness) lesions. When hypopyon was present, its height was measured in millimeters from the inferior corneal limbus.

For laboratory diagnosis, we performed the smear and culture for each eye with suspected infectious keratitis. Corneal ulcers were scraped using a sterile scalpel blade after application of 0.5% proparacaine hydrochloride (Alcaine, Alcon, Fort Worth, TX, USA) for anesthesia. A corneal-scraping specimen was taken from the margin and base of the ulcer and was placed onto glass slides for direct microscopic examination (Gram stain and 10% KOH smear) and also inoculated onto a variety of media. For the KOH smear, one drop of 10% KOH was added to the specimen and placed over the cover glass in the dermatology clinical laboratory, and the direct microscopic examination was performed by dermatologists. These included blood agar, chocolate agar, MacConkey agar, and Sabouraud dextrose agar plates. Samples were inoculated into Sabouraud plates for fungal detection and stored at room temperature for 28 days. Microorganisms were identified using standard laboratory techniques that have been reported previously [20].

When a fungal infection was clinically suspected, topical and systemic antifungal treatment was started immediately. Topical antifungal therapy consisted of hourly instillation of topical 5% natamycin, or 0.2% fluconazole, or/and 0.15% amphotericin B. Systemic antifungal agents were administered with oral fluconazole (50 mg·bid·p. o.) and intravenous amphotericin B. Topical 2% voriconazole was used in case of no clinical improvement. All patients were treated topically with 3^rd^ or 4^th^ generation fluoroquinolones either alone or in a combination with fortified topical aminoglycosides and intravenous systemic antibiotics as empirical therapy. Topical treatment was progressively tapered or modified according to the clinical response and bacterial susceptibility. In cases where initial medical treatment failed, various surgical treatments such as amniotic membrane transplantation, conjunctival flap, keratoplasty, and evisceration were performed. Treatment outcomes were assessed at the end of 3 months or at the completion of treatment. Poor clinical outcomes were defined as the increase in the size of a corneal ulcer or infiltrate, presence of corneal perforation, or when patients underwent penetrating keratoplasty or evisceration despite maximum medical therapy.

SPSS Statistics for Windows, version 23.0 (IBM Corp., Armonk, NY, USA) was used for statistical analysis. All data are presented as a mean ± standard deviation for continuous variables and number (%) for categorical variables. Statistical analysis for normality was performed using the Kolmogorov–Smirnov test. Intergroup analysis was completed using the Mann–Whitney *U* test for data that were not normally distributed. The chi-square test was used to analyze categorical variables. Pearson's correlation analysis was used to evaluate a correlation between poor clinical outcomes and variable clinical factors. A *p* value less than 0.05 was considered statistically significant.

## 3. Results

Seventy-nine eyes at two tertiary hospitals were identified with *Fusarium* keratitis during a 20-year study period. Thirty-nine patients (49.4%) were males, and 40 (50.6%) were females. The mean age was 67.1 ± 10.2 years (range: 41–85, median 69 years). The most frequently affected eyes were left eyes (58.2%). Sixty-one patients (77.2%) had residence in rural areas, and 52 patients (65.8%) were involved in agricultural occupation. There were no significant differences in sex, laterality, and occupational distribution between the KOH-positive and KOH-negative groups (Table 1).

**Figure 1:**
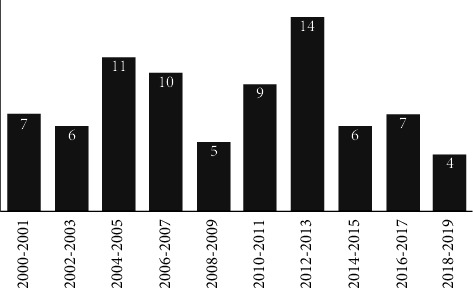
Incidence of *Fusarium* keratitis cases at two tertiary centers in South Korea during the period 2000–2019.

The biennial distribution of *Fusarium* keratitis is shown in Figure 1. The peak incidence of *Fusarium* keratitis occurred between 2005 and 2013. However, there was no significant trend reported over time. The mean time to symptom duration was 11.0 ± 15.7 days (range, 0–120 days). Accompanied systemic diseases included diabetes mellitus (15 patients, 19.0%) and hypertension (14 patients, 17.7%).

Various predisposing factors for *Fusarium* keratitis are identified and summarized in Table 2. The most common predisposing factor was ocular trauma (55.7%), followed by previous chronic ocular surface diseases (21.5%). There were no significant differences in notable predisposing factors between the two groups. Cases with no known predisposing factors were small in number in the KOH-positive group compared to the KOH-negative group (4 vs. 15, *p*=0.047). There were no contact lens wearers.

Among the clinical manifestations, the central location of corneal lesions was more common than the peripheral one in both groups. Also, ulcers with an irregular shape were more frequent than those with a circular shape in both groups. There were no significant differences in the location of corneal lesions, shape of ulcers, feathery margin, satellite lesions, hypopyon, and endothelial plaques between the two groups. However, there were a higher proportion of cases with deep corneal infiltration in the KOH-negative group (78.7%) than those in the KOH-positive group (50.0%) (*p*=0.008).

The mean pretreatment ulceration size was 11.1 ± 9.2 mm^2^. There were no significant differences in the size of corneal lesions between the two groups. Initial visual acuity was 1.58 ± 0.95 logMAR in the KOH-positive group, and it was 1.53 ± 0.80 in the KOH-negative group. However, the difference was not statistically significant. However, impaired final visual acuity was higher in the KOH-negative group (1.94 ± 1.25 logMAR) than that in the KOH-positive group (1.41 ± 1.28) (*p*=0.049) (Table 3). The delayed time to start initial antifungal eye drops was longer in the KOH-negative group (3.93 ± 4.89 days) than that in the KOH-positive group (1.13 ± 0.49 days) (*p*=0.002) (Table 3).

After treatment, the ratio of visual acuity change was a higher proportion of cases with improved visual acuity (43.8%) in the KOH-positive group compared to aggravated visual acuity (55.3%) in the KOH-negative group (Table 4).

Only the KOH-negative group combined infection. Of 47 KOH-negative ulcers, 11 (23.4%) had mixed infections with bacterial organisms. In the case of bacteria, Gram-positive bacteria were predominant (90.9% of all bacterial cultures). The most common bacterial species was *Staphylococcus epidermidis* (7 of 11; 63.6%), followed by *Streptococcus viridans* (2 of 11; 18.2%) (Table 5).

For topical antifungal treatment, amphotericin B eye drops were the most commonly used in both groups, followed by fluconazole and voriconazole. The use of topical amphotericin B and fluconazole was significantly higher in the KOH-positive group (96.9% vs. 68.1%, *p*=0.002; 62.5% vs. 31.9%, *p*=0.007, respectively). However, the use of topical natamycin was significantly higher in the KOH-negative group (3.1%, vs. 25.5%, *p*=0.008) (Table 6).

Many patients underwent various surgical interventions: evisceration in 13 eyes (16.5%), amniotic membrane transplantation in 8 eyes (10.1%), penetrating keratoplasty in 6 eyes (7.6%), conjunctival flap in 2 eyes (2.5%), and corneal button graft in 2 eyes (2.5%). The proportion of evisceration was significantly higher in the KOH-negative group (3.1% vs. 25.5%, *p*=0.008) (Table 6).

In a multivariate logistic regression analysis, central corneal lesions (odds ratio (OR) 3.500, 95% confidence interval (CI) 1.018–12.040, *p*=0.047), a large ulcer size (size ≥ 7.5 mm^2^) (OR 4.980, 95% CI 1.504–16.490, *p*=0.009), and endothelial plaque (OR 7.004, 95% CI 1.189–41.271, *p*=0.031) were identified as significant risk factors for poor clinical outcomes. Admission treatment (OR 8.227, 95% CI 0.892–75.868, *p*=0.063) was not significant in multivariate analysis (Table 7).

## 4. Discussion

Few studies have reported on clinical differences between KOH-positive and KOH-negative patients with *Fusarium* keratitis. In this study, 59.5% of patients were negative for the initial KOH smear prior to their final diagnosis of *Fusarium* keratitis. Cases with deep corneal infiltration and cases without significant predisposing factors were more frequently observed in the initial KOH-negative group. The time delay to use initial antifungal eye drops after the first visit was longer in the KOH-negative group. In cases of the initial KOH negative result, diagnosis of *Fusarium* keratitis was hindered until a definite culture result was obtained. Also, combined bacterial infection was reported only in the KOH-negative group. In these cases, fungal keratitis could not be considered due to the initial bacterial infection. Therefore, antifungal treatment was delayed, and evisceration was more frequently performed in the KOH-negative group in this study.

Geographical and seasonal factors play an important role in the distribution and epidemiology of fungal infection [9]. *Fusarium* spp. was found as the main causative microbes of fungal keratitis in tropical and subtropical areas such as Florida [21]. Two tertiary hospitals in this study located in Jeolla Province, south area of South Korea, subtropical area. Two hospitals were most frequently reported hospitals of fungal keratitis in South Korea, between 2008 and 2017 [22].

One study in the Netherlands between 2005 and 2016 reported a significant increase in the number of cases of *Fusarium* keratitis since 2010 [3]. However, in this study, there was no significant annual difference of the incidence of *Fusarium* keratitis between 2000 and 2019.

The main risk factor for *Fusarium* keratitis was a history of ocular trauma, mainly in people that have agriculture-related occupations [2, 9, 10, 23]. Our study confirms this information, finding 65.8% with agricultural occupation and 55.7% with a history of trauma, mostly soil and plant material.

With increasing contact lens wearing, *Fusarium* keratitis has also become a problem in urban areas with temperate climates. However, there were no patients associated with contact lens wearing in this study. One of the reasons may be that most of keratitis cases were reported in elderly patients with agricultural activity in rural areas, so they did not wear contact lenses.

The authors predicted that patients in the KOH-positive group would have more severe clinical manifestations than those in the KOH-negative group. However, this study found no significant differences in clinical characteristics between the two groups except only in terms of corneal infiltration depth. At initial examination, deep corneal stromal infiltration was common in the KOH-negative group. One potential explanation of this result is that mixed bacterial infection in the KOH-negative group might be more involved in deep corneal stromal infiltration. In a study by Ahn et al. [24], it was reported that mixed bacterial and fungal keratitis showed more common deep corneal stromal infiltration and poor prognosis. Therefore, they recommended that when treatment against the initial causative microbes of corneal ulcers fails, combined infection should be suspected and the repeated smear and culture should be considered.

Direct microscopic examination of a corneal scraping material is a rapid and available method for diagnosis of fungal keratitis [18]. Preparation of corneal scrapings with 10% KOH wet mount and trying to find filamentous fungi or yeast cells is a simple and inexpensive technique for diagnosis of fungal keratitis [25]. Bharathi et al. [26] investigated the diagnostic value of the 10% KOH smear for detection of fungal filaments on 3298 eyes. According to this study, the 10% KOH smear had noticeable importance with a sensitivity and specificity positive predictive value and negative predictive value of 99.3%, 99.1%, 98.5%, and 99.6%, respectively. In comparison to these findings, Badiee et al. [27] and Vengayil et al. [28] reported that the sensitivity and specificity of the KOH smear was 68% and 60%, respectively. Usually, the sensitivity of the KOH smear may be adversely affected by the insufficient amount of sampling materials, the small size of the corneal ulcer, and the lack of individual experience of the microscopic observer [26]. In this study, nearly 60% patients were initially KOH negative and finally culture positive. In the KOH-negative group, final visual acuity was worse than in the KOH-positive group, and also, evisceration treatment was performed more frequently. The delay of prompt initiation of antifungal therapy in the KOH-negative group may result in a poor prognosis. Therefore, although the KOH smear and culture are of variable sensitivity, they should be performed for all corneal ulcer patients as the basic diagnostic examination.

For the treatment of *Fusarium* keratitis, topical use of fluconazole and amphotericin B was significantly higher in the KOH-positive group in this study. Also, subconjunctival injection of an antifungal agent was higher in the KOH-positive group. Naturally, early diagnosis contributes to early treatment with topical and local injection of antifungal agents.

At presentation, there was no significant difference between the KOH-positive and KOH-negative groups in visual acuity. After treatment, improvement of visual acuity was more common in the KOH-positive group. On the contrary, cases with aggravation of visual acuity were more common in KOH-negative*Fusarium* keratitis. There was no statistically significant difference between the KOH-positive and KOH-negative groups in the change of visual acuity after treatment. However, at last follow-up, the KOH-negative group had significantly lower visual acuity. Pérez-Balbuena et al. [10] noted that final visual acuity after medical/surgical treatment was 64.9% patients with less hand movement (HM). They concluded that the outcome of *Fusarium* keratitis is generally poor in spite of various treatment options available.

We performed a logistic regression analysis to determine risk factors for poor clinical outcomes, in cases of such as progression, perforation, penetrating keratoplasty, and evisceration. As a result, central corneal lesions (OR 3.50, 95% CI 1.02–12.04, *p*=0.047), a large ulcer size (size ≥ 7.5 mm^2^) (OR 4.98, 95% CI 1.50–16.49, *p*=0.009), and endothelial plaque (OR 7.00, 95% CI 1.19–41.27, *p*=0.031) were highly associated with poor clinical outcomes. Lalitha et al. [29] reported that the presence of hypopyon was a significant predictor of treatment failure in fungal keratitis. Cho and Lee [30] stated significant risk factors for treatment failure were hypopyon and deep infiltration in microbiologically proven fungal keratitis. In this study about *Fusarium* keratitis, deep corneal infiltration and hypopyon were significant in univariate logistic regression analysis, but their effects were attenuated in multivariate regression analysis. Central lesion, large epithelial defect size, and endothelial plaque can be regarded as a marker of a poor prognosis.

This study has several limitations. First, the sample size was small. Second, this study was confined to Jeolla Province, South Korea, which is temperate to subtropical climates and with four seasons. Third, because the study was conducted by the tertiary hospital, the results of this study cannot be generalized. Fourth, only patients with microbiological proven *Fusarium* keratitis were enrolled in this study, while cases of other fungal or clinically suspected fungal keratitis were excluded. Therefore, the effect of the initial KOH smear in this study cannot be generalized on all fungal keratitis. Despite such limitations, this study has an important clinical significance. This investigation highlights the initial KOH effect on early diagnosis and early treatment of *Fusarium* keratitis. This study is a clinical analysis of *Fusarium* keratitis in areas with high fungal prevalence in South Korea. Clinicians may use these findings as a useful reference for various regional differences in *Fusarium* keratitis.

To our knowledge, this is the first study evaluating the clinical outcomes of *Fusarium* keratitis that divides two groups depending on the initial KOH smear result. In this study, the KOH-negative group had worse final visual acuity, and evisceration was performed frequently in the KOH-negative group than in the KOH-positive group. The delay of prompt initiation of antifungal treatment results in a poor prognosis. This study highlights the initial KOH effect on early diagnosis and early treatment of *Fusarium* keratitis.

## Figures and Tables

**Table 1:** Epidemiology for eyes with culture-proven*Fusarium* keratitis divided by the initial KOH smear.

Characteristics	KOH-positive (*n* = 32)	KOH-negative (*n* = 47)	Total (*n* = 79)	*p* value ^*∗*^
Sex				0.926
Male	16 (50.0)	23 (48.9)	39 (49.4)	
Female	16 (50.0)	24 (51.1)	40 (50.6)	
Laterality				0.865
Right eye	13 (40.6)	20 (42.6)	33 (41.8)	
Left eye	19 (59.4)	27 (57.4)	46 (58.2)	
Occupation				0.156
Agriculture	24 (75.0)	28 (59.6)	52 (65.8)	
Nonagriculture	8 (25.0)	19 (40.4)	27 (34.2)	
Residence				0.211
Urban	5 (15.6)	13 (27.7)	18 (22.8)	
Rural	27 (84.4)	34 (72.3)	61 (77.2)	
Systemic disease				
Hypertension	6 (18.8)	8 (17.0)	14 (17.7)	
Diabetes mellitus	3 (9.4)	12 (25.5)	15 (19.0)	
Other^†^	4 (12.5)	5 (10.6)	9 (11.4)	

Values are presented as a number (%). KOH = potassium hydroxide.  ^*∗*^A *p*, *p* value was calculated using the chi-square test; ^†^other: cerebral-cardiac vascular disease (3), Hansen's disease (2), liver cirrhosis (2), tuberculosis (1), and so on.

**Table 2:** Ocular predisposing factors for eyes with culture-proven*Fusarium* keratitis divided by the initial KOH smear.

Predisposing factors ^*∗*^	KOH-positive (*n* = 32)	KOH-negative (*n* = 47)	Total (*n* = 79)	*p* value^†^
Ocular trauma	19 (59.4)	25 (53.2)	44 (55.7)	0.587
Soil	9 (28.1)	12 (25.5)	21 (26.6)	
Plant	9 (28.1)	9 (19.1)	18 (22.8)	
Unknown	1 (3.1)	4 (8.5)	5 (6.3)	
Prior ocular surface disease	8 (25.0)	9 (19.1)	17 (21.5)	0.534
Corneal opacity	3 (9.4)	3 (6.4)	6 (7.6)	
Herpes simplex keratitis	1 (3.1)	3 (6.4)	4 (5.1)	
Scleromalacia	1 (3.1)	1 (2.1)	2 (2.5)	
Necrotizing scleritis	1 (3.1)	1 (2.1)	2 (2.5)	
Bullous keratopathy	1 (3.1)	0	1 (1.3)	
Eyelid abnormalities	1 (3.1)	0	1 (1.3)	
Exposure keratitis	0	1 (2.1)	1 (1.3)	
Previous ocular surgery	2 (6.3)	2 (4.3)	4 (5.1)	0.691
Topical or systemic use of steroids	5 (15.6)	5 (10.6)	7 (8.9)	0.513
Contact lens	0	0	0	
No significant history	4 (12.5)	15 (31.9)	19 (24.1)	0.047

Values are presented as a number (%). KOH = potassium hydroxide.  ^*∗*^Fifteen patients had 2 ocular risk factors simultaneously; ^†^a *p* value was calculated using the chi-square test.

**Table 3:** Clinical manifestations for eyes with culture-proven*Fusarium* keratitis divided by the initial KOH smear.

Variables	KOH-positive (*n* = 32)	KOH-negative (*n* = 47)	Total (*n* = 79)	*p* value ^*∗*^
Location				0.722
Central	14 (43.8)	23 (48.9)	37 (46.8)	
Paracentral	13 (40.6)	15 (31.9)	28 (35.4)	
Peripheral	5 (15.6)	9 (19.1)	14 (17.7)	
Shape of ulcers				0.467
Irregular	26 (81.2)	41 (87.2)	67 (84.8)	
Circle	6 (18.8)	6 (12.8)	12 (15.2)	
Depth of infiltration		`		0.008
Superficial	16 (50.0)	10 (21.3)	26 (32.9)	
Deep	16 (50.0)	37 (78.7)	53 (67.1)	
Feathery margin	13 (40.6)	16 (34.0)	29 (36.7)	0.551
Satellite lesion	4 (12.5)	2 (4.3)	6 (7.6)	0.174
Hypopyon	16 (50.0)	28 (59.6)	44 (55.7)	0.400
Endothelial plaque	3 (9.4)	10 (21.3)	13 (16.5)	0.161
Size of ulcers (mm^2^)	10.26 ± 8.75	11.58 ± 9.60	11.05 ± 9.24	0.444^†^
Initial VA (logMAR)	1.58 ± 0.95	1.53 ± 0.80	1.56 ± 0.86	0.698^†^
Final VA (logMAR)	1.41 ± 1.28	1.94 ± 1.25	1.72 ± 1.28	0.049^†^
Time to start initial antifungal eye drops	1.13 ± 0.49	3.93 ± 4.89	2.75 ± 3.97	0.002^†^

Values are presented as a mean ± standard deviation or number (%). KOH = potassium hydroxide, VA = visual acuity, LogMAR = logarithm of the minimum angle of resolution.  ^*∗*^Chi-square test; ^†^a *p* value was calculated using the Mann–Whitney test.

**Table 4:** The change of visual acuity after treatment in eyes with culture-proven*Fusarium* keratitis divided by the initial KOH smear.

VA	KOH-positive (*n* = 32)	KOH-negative (*n* = 47)	Total (*n* = 79)	*p* value ^*∗*^
Improved	14 (43.8)	13 (27.7)	27 (34.2)	0.107
No change	8 (25.0)	8 (17.0)	16 (20.3)	
Aggravated	10 (31.2)	26 (55.3)	36 (45.6)	

Values are presented as a number (%). VA = visual acuity; KOH = potassium hydroxide.  ^*∗*^Chi-square test.

**Table 5:** Combined bacterial distribution in the KOH-negative group.

	KOH-negative	Combined bacteria (number of isolates)
Gram-positive bacteria	10 (21.3)	*Staphylococcus epidermidis* (7), Viridans streptococcus (2), Gram-positive rod (1)
Gram-negative bacteria	1 (2.1)	*Burkholderia cepacia* (1)

Values are presented as a number (%). KOH = potassium hydroxide.

**Table 6:** Medical and surgical treatment for eyes with culture-proven*Fusarium* keratitis divided by the initial KOH smear.

Treatments	KOH-positive (*n* = 32)	KOH-negative (*n* = 47)	Total (*n* = 79)	*p* value^†^
Topical treatment ^*∗*^				
Amphotericin B	31 (96.9)	32 (68.1)	63 (79.7)	0.002
Fluconazole	20 (62.5)	15 (31.9)	35 (44.3)	0.007
Voriconazole	17 (53.1)	15 (31.9)	32 (40.5)	0.059
Natamycin	1 (3.1)	12 (25.5)	13 (16.5)	0.008
Moxifloxacin	21 (65.6)	24 (51.1)	45 (57.0)	0.199
Other treatment				
IC antifungal injection	9 (28.1)	10 (21.3)	19 (24.1)	0.484
SC antifungal injection	8 (25.0)	4 (8.5)	12 (15.2)	0.045
Surgical treatment				
PKP	3 (9.4)	3 (6.4)	6 (7.6)	0.622
Evisceration	1 (3.1)	12 (25.5)	13 (16.5)	0.008
Corneal button graft	1 (3.1)	1 (2.1)	2 (2.5)	0.782
AMT	4 (12.5)	4 (8.5)	8 (10.1)	0.564
Conjunctival flap	0	2 (4.3)	2 (2.5)	0.237

Values are presented as a number (%). KOH = potassium hydroxide, IC = intracameral, SC = subconjunctival, PKP = penetrating keratoplasty, AMT = amniotic membrane transplantation.  ^*∗*^Initially selected eye drops. ^†^Chi-square test.

**Table 7:** Risk factor for poor clinical outcomes in the total cohort of patients with culture-proven*Fusarium* keratitis (univariate and multivariate logistic regression analyses).

Variables	Univariate			Multivariate		
	Odds ratio	95% CI	*p* value ^*∗*^	Odds ratio	95% CI	*p* value ^*∗*^
Male sex	1.292	0.520–3.212	0.581			
Age (≥70 years)	1.362	0.546–3.397	0.507			
DM	0.900	0.285–2.843	0.858			
Agricultural occupation	0.574	0.213–1.549	0.273			
Ocular trauma	0.480	0.187–1.233	0.127			
Previous ocular surface disease	2.347	0.687–8.022	0.174			
Central corneal lesion	3.987	1.482–10.727	0.006	3.500	1.018–12.040	0.047
Deep stromal infiltration	0.289	0.109–0.772	0.013	—	—	—
Ulcer size ≥ 7.5 mm^2^	5.672	2.075–15.505	0.001	4.980	1.504–16.490	0.009
Initial BCVA < 0.02 (Snellen)	2.667	1.035–6.873	0.042	—	—	—
Hypopyon	2.252	0.892–5.689	0.086	—	—	—
Endothelial plaque	4.053	0.832–19.749	0.083	7.004	1.189–41.271	0.031
KOH stain positivity	0.424	0.167–1.079	0.072	—	—	—
Bacterial mixed infection	2.308	0.580–9.176	0.235			
Admission treatment	4.600	0.831–25.449	0.080	8.227	0.892–75.868	0.063

CI = confidence interval; DM = diabetes mellitus; BCVA = best corrected visual acuity; KOH = potassium hydroxide.  ^*∗*^Multivariate logistic regression analysis was performed using the backward conditional method for the factors with a *p* value <0.1 in univariate logistic regression analysis.

## Data Availability

The detailed data used to support the findings of this study are available from the corresponding author upon request.
